# Diagnostic Testing After Positive Cell‐Free DNA Screening for Sex Chromosome Aneuploidies: Clinical and Socioeconomic Determinants

**DOI:** 10.1002/pd.70229

**Published:** 2026-08-01

**Authors:** Blair K. Stevens, Sarah Araji, Mohamad Ali Maktabi, Randy Liu, Lauren Westerfield, Michael Wassef, Dejian Lai, Ignatia B. Van den Veyver, Meera Krishnan, Charlotte Lawrence, Siddharth K. Prakash

**Affiliations:** ^1^ Department of Obstetrics, Gynecology, and Reproductive Sciences University of Texas Health Science Center at Houston Houston Texas USA; ^2^ Department of Obstetrics and Gynecology Baylor College of Medicine Houston Texas USA; ^3^ Department of Molecular and Human Genetics Baylor College of Medicine Houston Texas USA; ^4^ John P and Kathrine G McGovern Medical School The University of Texas Health Science Center at Houston Houston Texas USA; ^5^ Department of Biostatistics and Data Science The University of Texas Health Science Center at Houston School of Public Health Houston Texas USA; ^6^ Department of Internal Medicine The University of Texas Health Science Center at Houston Houston Texas USA

**Keywords:** cell‐free DNA screening, health disparities, prenatal diagnosis, sex chromosome aneuploidy, socioeconomic factors

## Abstract

**Objective:**

To assess socioeconomic and medical factors associated with prenatal confirmatory diagnostic testing after positive prenatal cell‐free (cfDNA) screening for sex chromosome aneuploidies (SCA) in a diverse contemporary patient cohort.

**Methods:**

We conducted a retrospective cohort study of patients who had abnormal prenatal cfDNA screening results indicative of SCA and were seen for genetic counseling between 2014 and 2024 at two tertiary academic medical centers. We used logistic regression analysis to evaluate the association of demographic and clinical factors with confirmatory testing.

**Results:**

Of the 692 patients with abnormal cfDNA results, 26% (*n* = 180) underwent diagnostic testing. These patients were older, resided in more affluent areas, presented earlier for genetic counseling, were more likely to have commercial insurance, and were less likely to prefer Spanish or use an interpreter. Compared to Non‐Hispanic (NH) Whites, diagnostic testing rates were higher for NH Asians but were lower for NH Blacks and Hispanics or when results indicated XYY or XXX. In logistic modeling, lower deprivation scores and commercial insurance were independently associated with higher testing rates.

**Conclusion:**

Only 1 in 4 patients pursued prenatal diagnostic testing following positive cfDNA screening for SCAs. Race, ethnicity, socioeconomic status and preferred language were the primary determinants of testing.

## Summary

- What is already known about this topic?
  ◦ cfDNA screening has expanded the detection of sex chromosome aneuploidies, but confirmatory diagnostic testing rates vary following a positive result.
- What does this study add?
  ◦ In this cohort, 26% underwent confirmatory testing.
  ◦ Uptake was strongly associated with insurance type and neighborhood deprivation, preferred language, interpreter use, and race/ethnicity.
  ◦ Insurance coverage alone does not ensure equitable access. Structural barriers likely influence decision‐making, highlighting the need for targeted counseling, care navigation, and pathways for postnatal diagnosis.

**Abbreviations**
cfDNA: cell‐free DNA
GC: genetic counseling
SCA: sex chromosome aneuploidy

## 1 Background

Sex chromosome aneuploidies (SCAs), also referred to as X and Y chromosome variations, arise from the presence of an extra or missing sex chromosome. Common SCAs include 45,X (Turner syndrome), 47, XXY (Klinefelter syndrome), 47, XXX (trisomy X), and 47, XYY (Jacobs syndrome). Phenotypic expression is highly variable, ranging from significant congenital anomalies to subtle or nonspecific findings. While monosomy X may be suspected prenatally due to ultrasound findings such as cystic hygroma or congenital heart disease [1], most SCAs are not detected on routine anatomy ultrasound. Consequently, 80%–90% of individuals with SCAs remain undiagnosed postnatally [2].

Cell‐free DNA (cfDNA) screening for SCAs was introduced in 2012, with high sensitivity (> 95%) and specificity (> 99%) [3]. However, cfDNA remains a screening modality, and positive predictive values vary substantially; in some cohorts, more than half of positive results are false positives [4]. Accordingly, professional societies recommend confirmatory diagnostic testing via chorionic villus sampling (CVS) or amniocentesis following a positive cfDNA result [5].

Despite these recommendations, many patients decline diagnostic testing [6, 7]. Decision‐making in this context is complex and requires genetic counseling regarding test performance, natural history, phenotypic variability, procedural risks, the lack of in‐utero treatments, and reproductive management options, or lack thereof. For example, access to abortion care for fetal indications in Texas is restricted [8, 9, 10].

The decision to undergo prenatal diagnostic testing should align with patient values and pregnancy goals. Some patients do not desire a prenatal diagnosis because it would not change their pregnancy management for religious or spiritual reasons, or because they were not comfortable with the risks associated with CVS and amniocentesis [11]. If prenatal testing is declined, postnatal testing is necessary because early diagnosis enables anticipatory management of associated conditions, including congenital heart disease, endocrine dysfunction, renal anomalies, and neurodevelopmental differences. Interventions, such as cardiac screening, hormone replacement therapy, and early childhood intervention, can improve outcomes and may mitigate downstream disparities in care [12, 13, 14]. Additionally, given the high rate of false positive screening results, diagnostic testing is critical in determining who may not need additional screening and interventions.

More patients are confronted with potential SCA diagnoses during pregnancy due to widespread utilization of cfDNA screening; however, it remains unclear which factors influence completion of confirmatory testing. Barriers to diagnostic testing, either before or after birth, likely operate at multiple levels, including access to specialty care, health system navigation, and social determinants of health such as race/ethnicity, insurance status, language, and geographic proximity to care [15, 16]. While lower rates of prenatal diagnostic testing uptake may reflect structural barriers to care, they are also related to patient‐centered decision‐making based on individual values, preferences, perceptions of risk, and anticipated utility of prenatal diagnostic information.

In this multi‐center retrospective study, we aimed to assess socioeconomic and medical factors associated with prenatal confirmatory diagnostic testing after positive cfDNA screening for SCAs in a diverse contemporary patient cohort.

## 2 Methods

We conducted a retrospective cohort study of patients with abnormal prenatal cfDNA results suggestive of SCAs who received genetic counseling between 2014 and 2024 at two tertiary academic centers in Harris County, Texas. The protocol for this study was approved by the University of Texas Health Science Center at Houston IRB (HSC‐MS‐24‐1271) on 1/16/25 and by the Baylor College of Medicine IRB (H‐57019) on 02/11/2025. The requirement for informed consent was waived by both IRBs; therefore, a patient consent statement is not applicable or needed for this study. Data from eligible patients were collected and stored in REDCap, a secure, HIPAA compliant, web‐based software platform designed to support data capture for research studies. The system features user authentication and role‐based access controls to protect patient privacy. All PHI except genetic counseling consult date and parental age (years) were removed prior to analysis.

Both institutions provide care through multiple urban and suburban maternal‐fetal medicine clinics, with access to in‐person and virtual genetic counseling. Clinical databases maintained by genetic counselors were queried for patients with cfDNA results indicating monosomy X, XXY, XYY, or XXX. Patients referred for abnormal cfDNA results or identified through genetic counseling encounters were included. Cases involving atypical sex chromosome findings, autosomal aneuploidies, low fetal fraction, or non‐reportable results were excluded. Missing data were supplemented through electronic medical record review.

Extracted variables included cfDNA result, diagnostic testing uptake and outcomes, maternal and paternal demographics, preferred language, interpreter use, gestational age at counseling, gravidity, insurance status, ultrasound findings, family history, and Area Deprivation Index (ADI) derived from census‐based measures of socioeconomic status. Of note, maternal age was recorded in the clinical databases and collected for this study as “maternal age at delivery” using a calculation based on the patient's date of birth and due date. Age is recorded in this way for maternal age‐based risk assessment purposes. However, not all pregnancies in this cohort resulted in delivery as some pregnancies were terminated or were lost to miscarriage.

The primary outcome was completion of prenatal diagnostic testing via CVS or amniocentesis. Odds ratios (ORs) with 95% confidence intervals (CIs) were calculated. Multivariable logistic regression models were constructed using clinically relevant variables. Separate models were used for clinical predictors (e.g., ultrasound findings, SCA type, family history), and demographic variables. Firth penalized likelihood methods were applied where standard maximum likelihood estimation did not converge. Statistical significance was defined as *p* < 0.05.

Patients classified as multiracial/other or with rare categories were excluded from regression analyses because of small sample sizes. Sensitivity analyses confirmed that exclusions did not alter the results.

## 3 Results

**FIGURE 1:**
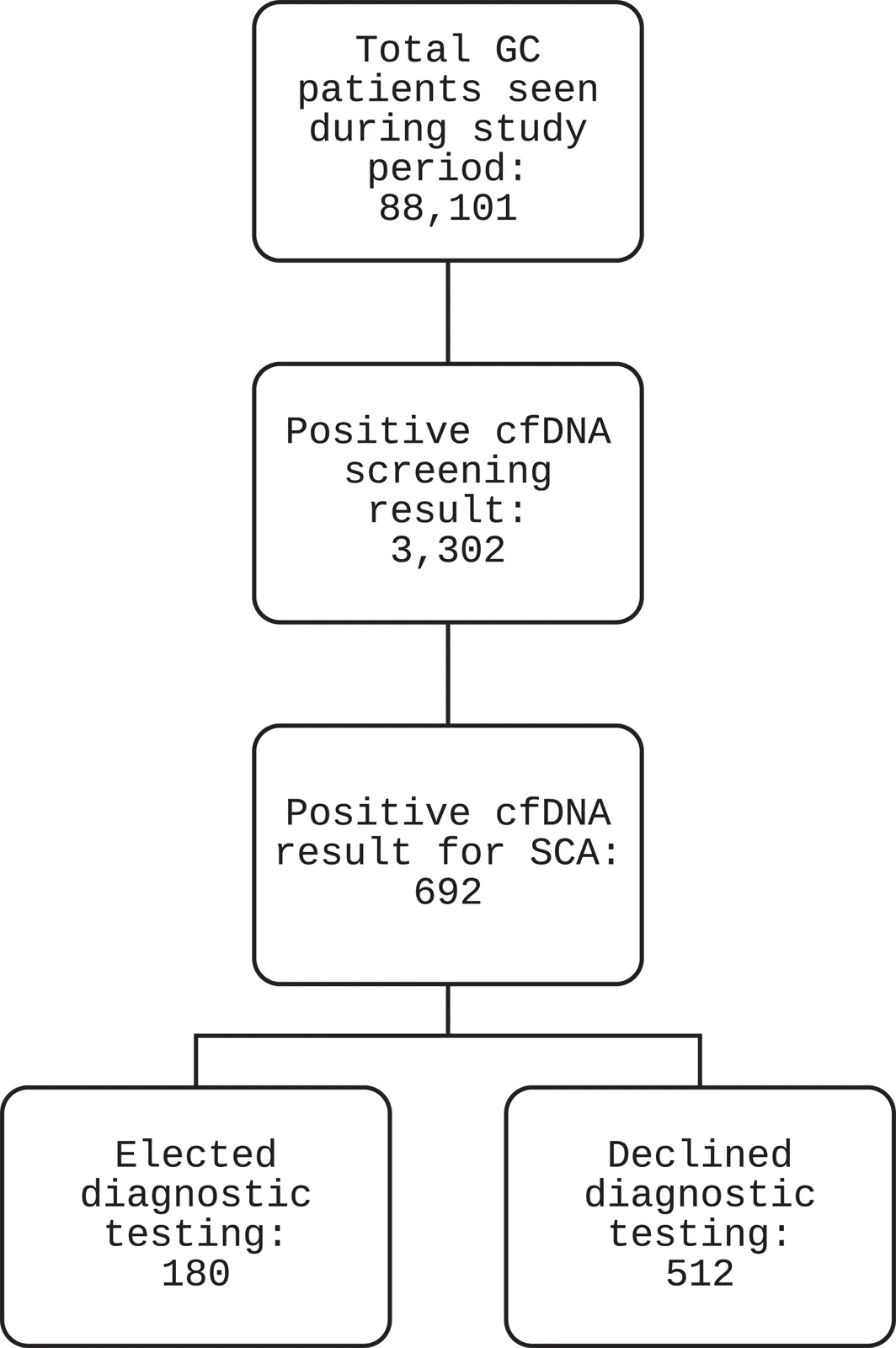
CONSORT diagram illustrating case selection for study.

Among 88,101 patients receiving genetic counseling, 3302 had abnormal cfDNA results, including 692 (21%) with SCAs (Figure 1). The cohort was 37% Hispanic, 33% non‐Hispanic White, 16% non‐Hispanic Black, and 13% non‐Hispanic Asian; 73% were multigravida, 89% were English‐speaking, and 59% had commercial insurance. cfDNA results included monosomy X (*n* = 353, 51%), XXY (*n* = 150, 22%), XYY (*n* = 85, 12%), and XXX (*n* = 93, 13%). Ultrasound abnormalities were present in 29%, and 18% reported a relevant family history (personal history of stillbirth or recurrent miscarriages, previous child/pregnancy with birth defect or personal or family history of genetic disorder) (Table 1).

**TABLE 1:** Demographic and clinical characteristics stratified by fetal diagnostic testing.

	N with data available	Total mean (SD) or *n* (%)	Declined testing *n*, mean or *n* (%)	Elected Testing *n*, mean	
Maternal age at delivery (years)	692	31 (6)	512, 31	180, 32	**0.0044**
Maternal race/Ethnicity *N* (%)	687				**< 0.0001**
NH white		230 (33)	162 (32)	68 (38)	
NH black		108 (16)	88 (17)	20 (11)	
NH asian		88 (13)	49 (10)	39 (22)	
Hispanic		253 (37)	207 (41)	46 (26)	
Other/multiracial		8 (1)	3 (1)	5 (3)	
Paternal age at delivery (years)	386	33 (7)	292, 33	94, 34	0.4125
Paternal race/Ethnicity *N* (%)	446				**< 0.0001**
NH white		159 (36)	104 (31)	55 (49)	
NH black		73 (16)	59 (18)	14 (13)	
NH asian		51 (12)	31 (9)	20 (18)	
Hispanic		1498 (33)	129 (39)	20 (18)	
Other		14 (3)	11 (3)	3 (3)	
Gravity *N* (%)	692				0.9406
Primigravida		186 (27)	138 (27)	48 (27)	
Multigravida		506 (73)	374 (73)	132 (73)	
Preferred language *N* (%)	691				**0.0064**
English		614 (89)	447 (87)	167 (93)	
Spanish		57 (8)	52 (10)	5 (3)	
Other		20 (3)	13 (3)	77 (4)	
Interpreter *N* (%)	690				**0.0222**
Interpreter not utilized		621 (90)	452 (88)	169 (94)	
Interpreter utilized		69 (10)	59 (12)	10 (6)	
Average state ADI (%)	690	48 (24)	510, 51	180, 40	**0.0001**
Insurance *N* (%)	683				
Uninsured		6 (1)	6 (1)	0 (0)	
Government insurance only		259 (38)	217 (43)	42 (24)	
Commercial insurance only		405 (59)	271 (54)	134 (75)	
Other coverage		13 (2)	11 (2)	2 (1)	
Gestational age at first GC visit (weeks)	692	17 (5)	512, 17	180, 15	**0.0001**
cfDNA screening high risk result N (%)	692				**0.0187**
Monosomy X		353 (51)	249 (49)	104 (58)	
XXY		150 (22)	107 (21)	43 (24)	
XYY		85 (12)	70 (14)	15 (8)	
XXX		93 (13)	75 (15)	18 (10)	
Other SCA		11 (2)	11 (2)	0 (0)	
Ultrasound findings *N* (%)	665				0.0777
Normal ultrasound		469 (71)	335 (69)	134 (76)	
Abnormal ultrasound		196 (29)	153 (31)	43 (24)	
Family history *N* (%)	666				0.5154
Not significant		546 (82)	407 (83)	139 (80)	
Significant		120 (18)	86 (17)	34 (20)	

*Note:* Demographic and clinical characteristics of patients with abnormal cfDNA screening for SCAs, stratified by whether fetal diagnostic testing (amniocentesis or CVS) was performed. Categorical variables are reported as frequencies and percentages; continuous variables are reported as means (standard deviations). *p*‐values are based on two sample *t* test for continuous variables and chi square test for categorical variables. Bold values indicate a statistically significant difference.

Abbreviations: ADI: area deprivation index; cfDNA: cell‐free DNA; GC: genetic counseling; NH: non‐Hispanic; SCA: sex chromosome aneuploidy.

Overall, 26% (180/692) underwent diagnostic testing. Patients who completed testing were older (32 vs. 31 years, *P* = 0.004), presented earlier (15 vs. 17 weeks, *p* < 0.001), and resided in less deprived areas (ADI percentile 40 vs. 51, *p* < 0.001).

Diagnostic testing was more likely among patients with commercial insurance (OR 2.56, 95% CI 1.73–3.77, *p* < 0.01) and less likely among those preferring Spanish (OR 0.26, 95% CI 0.10–0.66, *p* < 0.01) or requiring an interpreter (OR 0.45, 95% CI 0.23–0.91, *p* = 0.03). Compared with non‐Hispanic White patients, diagnostic testing rates were higher among non‐Hispanic Asians (OR 1.99, *p* < 0.01) and lower among non‐Hispanic Black (OR 0.54, *P* = 0.03) and Hispanic patients (OR 0.51, *p* < 0.01). Similar patterns were observed based on partner race/ethnicity. Testing rates were lower for XYY (OR 0.51, *P* = 0.03) and XXX (OR 0.57, *P* = 0.05) compared with monosomy X. Ultrasound abnormalities (*p* = 0.08), family history (*p* = 0.52), gravidity (*p* = 0.94), and partner age (*p* = 0.41) were not significantly associated with testing uptake. In multivariable analysis, lower ADI (OR 1.01 per percentile decrease, *p* = 0.05) and commercial insurance (OR 2.37, *p* = 0.03) remained independently associated with higher diagnostic testing rates (Table 2).

**TABLE 2:** Logistic regression analysis of factors associated with diagnostic testing.

	Simple		Multiple	
	OR (95% CI)	*p*	OR (95% CI)	*p*
Maternal age at delivery (years)	1.04 (1.013, 1.073)	< 0.01	0.99 (0.92, 1.07)	0.85
Maternal race/Ethnicity				
NH White	(Reference)		(Reference)	
NH Black	0.54 (0.30, 0.93)	**0.03**	0.46 (0.10, 2.02)	0.11
Hispanic	0.51 (0.34, 0.80)	**< 0.01**	1.53 (0.67, 3.48)	0.38
NH Asian	1.99 (1.19, 3.33)	**< 0.01**	2.38 (0.67, 7.80)	0.15
Paternal age at delivery (years)	1.02 (0.98, 1.05)	0.41	0.99 (0.93, 1.03)	0.84
Paternal race/Ethnicity				
NH White	(Reference)		(Reference)	
NH Black	0.45 (0.23, 0.89)	0.02	1.72 (0.43, 6.70)	0.15
Hispanic	0.29 (0.17, 0.52)	**< 0.01**	0.45 (0.21, 1.00)	0.09
NH Asian	1.22 (0.64, 2.34)	0.55	0.54 (0.15, 1.96)	0.42
Insurance				
Government insurance only	(Reference)		(Reference)	
Commercial insurance only	2.56 (1.73, 3.77)	**< 0.01**	2.37 (1.03, 5.45)	**0.03**
Average state ADI (%)	0.98 (0.97, 0.99)	**< 0.01**	0.99 (0.97, 1.00)	**0.05**
Preferred language				
English	(Reference)		(Reference)	
Spanish	0.26 (0.10, 0.66)	**< 0.01**	0.17 (0.02, 19.91)	0.46
Interpreter				
Interpreter not utilized	(Reference)		(Reference)	
Interpreter utilized	0.45 (0.23, 0.91)	**0.03**	4.87 (0.002, 489.97)	0.50
Gravity *N* (%)				
Primigravida	(Reference)		(Reference)	
Multigravida	1.02 (0.69, 1.49)	0.94	0.60 (0.33, 1.10)	0.10
Gestational age at first GC visit (weeks)	0.91 (0.87, 0.95)	**< 0.01**	0.96 (0.90, 1.03)	0.23
cfDNA screening high risk for				
Monosomy X	(Reference)		(Reference)	
XXY	0.96 (0.63, 1.47)	0.86	0.83 (0.54, 1.30)	0.20
XYY	0.51 (0.28, 0.94)	**0.03**	0.47 (0.25, 0.88)	0.13
XXX	0.57 (0.32, 0.1.01)	**0.05**	0.51 (0.29, 0.92)	0.26
Family history *N* (%)				
Not significant	(Reference)		(Reference)	
Significant	1.16 (0.76, 1.80)	0.52	1.14 (0.73, 1.78)	0.57
Ultrasound abnormality				
Absent	(Reference)		(Reference)	
Present	0.70 (0.47, 1.04)	0.08	0.67 (0.45, 1.02)	0.06

*Note:* Logistic regression was used to evaluate predictors of fetal diagnostic testing for patients with positive cfDNA screening for SCAs. Simple odds ratios (ORs) with 95% confidence intervals (CIs) are shown for all candidate variables. Variables were selected based on clinical relevance with two separate data sets. The smaller dataset contains ultrasound abnormalities, the high risk cfDNA condition and family history. The larger dataset contains demographic variables. The classic MLE method failed to converge for larger dataset, so Firth method was used. Statistical significance was defined as *p* < 0.05. Bold values indicate a statistically significant difference.

Abbreviations: ADI: area deprivation index; cfDNA: cell‐free DNA; GC: genetic counseling; NH: non‐Hispanic; SCA: sex chromosome aneuploidy.

Among 180 patients undergoing diagnostic testing, 102 (57%) had abnormal results. Of the 102 abnormal results, 75 (73.5%) were concordant with the cfDNA results and non‐mosaic, 18 (17.6%) were concordant with the cfDNA results but with evidence of mosaicism, four (3.9%) were positive for a different sex chromosome related abnormality (ex. pentasomy X, del(X) (p11.4)), and five (4.9%) were positive for non‐SCA related findings. The non‐mosaic concordant, mosaic concordant and other abnormalities related to a sex chromosome were categorized as “true positives” (*n* = 97, 95.1%). The positive predictive value was higher for XYY (OR 12.62), XXY (OR 11.97), and XXX (OR 6.31) compared with monosomy X (all *p* < 0.01) (Table 3). No demographic or clinical variables were significantly associated with abnormal diagnostic testing results in multivariable analyses (Table S1).

**TABLE 3:** Positive predictive values.

cfDNA condition	Total *n*	True positives *n* (%)	OR (95% CI)	*p*
Monosomy X	353	34 (9.6)	(Reference)	(Reference)
XXY	150	37 (24.67)	11.97 (4.60–31.60)	*p* < 0.01
XYY	85	13 (15.29)	12.62 (2.69–59.16)	*p* < 0.01
XXX	93	13 (14.0)	6.31 (1.91–20.83)	*p* < 0.01

*Note:* Number of true positives stratified by cfDNA SCA condition, reported as frequencies and percentages. Simple odds ratios (ORs) with 95% confidence intervals (CIs) are shown with *p*‐values based on chi square test.

Abbreviation: cfDNA, cell‐free DNA.

Maternal testing was performed in 36 patients (2 aneuploidy FISH, 34 chromosome analyses, 2 CMAs) and abnormalities were identified in six cases, including five with mosaic monosomy X and one with an unbalanced X; Y translocation.

## 4 Discussion

In this multicenter cohort, socioeconomic factors, particularly insurance type and neighborhood deprivation, were the strongest predictors of diagnostic testing following a positive cfDNA screen for an SCA. Patients with commercial insurance and those residing in less deprived areas were significantly more likely to undergo confirmatory testing.

These findings are notable given that government and commercial insurance payors in Texas typically cover prenatal diagnostic procedures and fetal chromosome analysis. Furthermore, all patients in this study received genetic counseling, which included a discussion of insurance coverage and potential out of pocket costs. These results suggest that financial coverage alone does not ensure equitable access. Insurance status and area deprivation scores were investigated as they can serve as a proxy for disparities in resources, which are difficult to ascertain through a retrospective chart review. Structural and logistical factors, including transportation, work flexibility, childcare, and health system navigation, likely contribute to disparities in testing uptake. Although genetic counseling is increasingly accessible via telehealth, diagnostic procedures require in‐person visits and post‐procedure restrictions, which may disproportionately affect patients with fewer resources.

Language and race/ethnicity were also associated with testing uptake. Potential barriers related to health literacy, language and communication include difficulties among patients with limited English proficiency in understanding complex genetic information and interpreting test results [17, 18]. Additionally, cultural beliefs, values, and social contexts may influence perceptions of prenatal testing and reduce the perceived value or acceptability of prenatal detection [19]. The findings related to patient race/ethnicity are consistent with broader literature demonstrating disparities in prenatal care utilization and access to genetic services, which may be related to system‐level barriers, mistrust, or lower awareness of testing options [17, 20, 21, 22].

Widespread utilization of cfDNA screening has increased the detection of SCAs; however, the pathway from screening to prenatal diagnosis remains inequitable. Additional studies are needed to determine if these testing trends persist after birth. Delayed or absent diagnosis may exacerbate disparities in downstream care as early identification enables proactive management of structural cardiac and renal malformations, initiation of hormone replacement therapies where indicated, and early interventions for developmental delays, ADHD and autism [23, 24, 25, 26]. Additionally, underrepresentation of diverse populations in SCA research cohorts may be perpetuated by disparities in diagnostic confirmation [27].

Several implications emerge from these findings. First, clinicians should recognize that insurance coverage does not eliminate barriers to diagnostic testing. Second, enhanced care navigation and support services may improve access, particularly for patients facing logistical challenges. Third, qualitative studies are needed to better understand patient decision‐making, including perceptions of procedural risk and the clinical utility of diagnosis that may be unrelated to barriers to prenatal diagnosis, particularly in underserved populations such as low‐income and non‐English speaking patients and those who decline prenatal diagnostic testing. Finally, systems to facilitate postnatal diagnostic evaluation are essential for patients who decline prenatal testing.

## 5 Limitations

The retrospective study design and reliance on chart reviews preclude causal inferences and may not capture all relevant variables that influence decision‐making. Insurance status and ADI are imperfect proxies for individual socioeconomic status. Additionally, findings from two institutions within a single metropolitan area in Texas, which has restrictive abortion policies, may limit generalizability.

## 6 Conclusion

Among patients with a positive cfDNA screen for an SCA, socioeconomic factors were independently associated with the uptake of diagnostic testing. These differences in uptake persisted despite the broad availability of genetic counseling and procedural coverage. Efforts to improve equity in prenatal diagnosis must address structural and social determinants that influence access to confirmatory testing.

## Funding

The authors have nothing to report.

## Conflicts of Interest

IBV is an associate editor for Prenatal Diagnosis. SKP is funded by a grant from the National Center for Advancing Translational Sciences (1U54HD121580) related to sex chromosome aneuploidy research; however, this grant did not directly support the work presented in this manuscript. The other authors report no other conflicts of interest.

## Supporting information


**Table S1:** logistic regression analysis of factors associated with abnormal fetal diagnostic testing results.

## Data Availability

The data that support the findings of this study are available from the corresponding author upon reasonable request.
