# Supplementary material for: Diagnostic Testing After Positive Cell‐Free DNA Screening for Sex Chromosome Aneuploidies: Clinical and Socioeconomic Determinants
Source: Prenat Diagn. 2026 Aug 1;46(10):1529–35. doi: 10.1002/pd.70229 (PMC13571795; doi:10.1002/pd.70229)
Supplement: Supplementary file 1 [file PD-46-1529-s001.docx]

# **Supplemental Table 1.** Logistic Regression Analysis of Factors Associated with Abnormal Fetal Diagnostic Testing Results

|  | Simple | | Multiple | |
| --- | --- | --- | --- | --- |
|  | **OR (95% CI)** | ***P*** | **OR (95% CI)** | ***P*** |
| Maternal age at delivery (years) | 1.01 (0.96, 1.07) | **0.66** | 0.95  (0.81, 1.13) | 0.58 |
| Maternal Race/Ethnicity |  |  |  |  |
| NH White | (reference) |  | (reference) |  |
| NH Black | 1.09  (0.40, 2.96) | 0.87 | 3.73  (0.12, 110.33) | 0.61 |
| Hispanic | 1.41  (0.65, 3.05) | 0.38 | 1.12  (0.25, 5.13) | 0.46 |
| NH Asian | 1.93  (0.84, 4.43) | 0.12 | 3.34  (0.15, 79.60) | 0.65 |
| Paternal age at delivery (years) | 1.00  (0.94, 1.08) | 0.90 | 1.08  (0.93, 1.25) | 0.34 |
| Paternal Race/Ethnicity |  |  |  |  |
| NH White | (reference) |  | (reference) |  |
| NH Black | 0.72 (0.33, 1.58) | 0.42 | 1.44  (0.08, 23.96) | 0.63 |
| Hispanic | 0.42 (0.21, 0.85) | **0.02** | 1.81  (0.39, 8.44) | 0.29 |
| NH Asian | 1.38  (0.65, 2.97) | 0.40 | 0.20  (0.00, 5.11) | 0.24 |
| Insurance |  |  |  |  |
| Government insurance only | (reference) |  | (reference) |  |
| Commercial insurance only | 0.67  (0.33, 1.36) | 0.26 | 0.25  (0.04, 1.59) | 0.14 |
| Average State ADI (%) | 1.01  (0.99, 1.02) | 0.40 | 0.99  (0.97, 1.02) | 0.58 |
| Preferred language |  |  |  |  |
| English | (reference) |  | (reference) |  |
| Spanish | 9.20  (0.38, 222.7)) | 0.17* | 2.91  (0.05, 165.39) | 0.61 |
| Interpreter |  |  |  |  |
| Interpreter not utilized | (reference) |  | (reference) |  |
| Interpreter utilized | 17.60  (0.88, 350.7) | 0.06* | 1.00,1.00, 1.00)- | -,+ |
| Gravity N (%) |  |  |  |  |
| Primigravida | (reference) |  | (reference) |  |
| Multigravida | 0.81  (0.41, 1.59) | 0.54 | 0.36  (0.11, 1.17) | 0.09 |
| Gestational age at first GC visit (weeks) | 1.06  (0.97, 1.16) | 0.22 | 0.98 (0.83, 1.16) | 0.83 |
| cfDNA screening high risk for |  |  |  |  |
| Monosomy X | (reference) |  | (reference) |  |
| XXY | 10.71  (4.14, 27.71) | <0.01 | 0.83  (0.54, 1.30) | 0.42 |
| XYY | 11.29  (2.42,52.73) | **<0.01** | 0.47  (0.25, 0.88) | **0.02** |
| XXX | 6.08  (1.87,19.80) | **<0.01** | 0.51  (0.29, 0.92) | **0.02** |
| Family History N (%) |  |  |  |  |
| Not significant | (reference) |  | (reference) |  |
| Significant | 0.62  (0.29,1.31) | 0.21 | 1.13  (0.72, 1.77) | 0.59 |
| Ultrasound Abnormality |  |  |  |  |
| Absent | (reference) |  | (reference) |  |
| Present | 1.84  (0.89,3.79) | 0.10 | 0.69  (0.46, 1.03) | 0.07 |

Results from logistic regression analyses evaluating predictors of fetal diagnostic testing for patients with positive cfDNA screening for SCAs. Simple odds ratios (ORs) with 95% confidence intervals (CIs) are shown for all candidate variables. Variables included in the multiple logistic regression model were selected based on clinical relevance with two separate data sets. The smaller dataset contains ultrasound abnormalities, the high risk cfDNA condition and family history. The larger dataset contains demographic variables. The classic MLE methods failed to converge for the larger dataset, so Firth method was used. Statistical significance was defined as *P*<0.05. NH: non-Hispanic; ADI: area deprivation index; GC: genetic counseling; cfDNA: cell-free DNA; SCA: sex chromosome aneuploidy. *Firth method for zero cell. +, no estimate provided by SAS.
